# Risk factors for cranial cruciate ligament rupture in dogs participating in canine agility

**DOI:** 10.1186/s12917-022-03146-2

**Published:** 2022-01-15

**Authors:** Debra C. Sellon, Denis J. Marcellin-Little

**Affiliations:** 1 Department of Veterinary Clinical Sciences, College of Veterinary Medicine, Washington State University, PO Box 646610, Pullman, WA 99164-6610 USA; 2 Department of Surgical and Radiological Sciences, School of Veterinary Medicine, University of California, Davis, CA USA

**Keywords:** Dog, Canine agility, Cranial cruciate ligament, Questionnaire, Sports medicine, Rehabilitation, Conditioning

## Abstract

**Background:**

Cranial cruciate ligament rupture (CCLR) is one of the most common causes of pelvic limb lameness in dogs. Risk factors for CCLR include breed (especially large and giant breeds), body weight, gender and spay/neuter status, and age. Few studies have evaluated physical activity and fitness indicators, however, as risk factors for disease. This study used an online questionnaire distributed primarily via social media to assess risk factors for CCLR in dogs actively engaged in agility training or competition to determine demographic and physical activity factors associated with rupture.

**Results:**

Data from 260 dogs with CCLR were compared to similar data from 1006 dogs without CCLR. All dogs were actively training or competing in agility at the time of CCLR or the time of data submission, respectively. Physical characteristics associated with increased risk of CCLR included younger age, spayed female sex, greater body weight, and greater weight to height ratio. Agility activities associated with increased odds ratios included competition in events sponsored by the North American Dog Agility Council (NADAC), competing at novice and intermediate levels, and competing in fewer than 10 events/year. Odds ratios were lower in dogs that competed in events sponsored by United Kingdom Agility International (UKI). Other activities associated with increased odds ratio for CCLR included involvement in flyball activities and short walks or runs over hilly or flat terrain on a weekly basis. Activities associated with decreased odds ratio included involvement in dock diving, barn hunt, nosework, or lure coursing/racing activities and participation in core balance and strength exercises at least weekly.

**Conclusions:**

These results are consistent with previous studies demonstrating that body weight and spay/neuter status are risk factors for CCLR in dogs. This is the first report to demonstrate that risk of CCLR in agility dogs is decreased in dogs that engage in regular core strengthening exercises, compete more frequently, compete at higher levels, and compete in more athletically challenging venues.

**Supplementary Information:**

The online version contains supplementary material available at 10.1186/s12917-022-03146-2.

## Background

Cranial cruciate ligament rupture (CCLR) is one of the most common causes of pelvic limb lameness in dogs. The incidence of CCLR more than doubled between 1964 and 2003 [1] and it is estimated that dog owners in the United States spend more than one billion dollars annually for medical and surgical management [2]. In most affected dogs CCLR is considered to be the result of progressive degeneration of the ligament rather than acute trauma. Because of the many anatomic, genetic, and environmental factors thought to contribute to risk of CCLR, it has been difficult to develop preventive strategies [3]. Previously identified risk factors for CCLR include age, sex, neuter status, breed, and body weight [1, 4–11]. Little information is available about the influence of physical activity or athletic conditioning on risk of CCLR. One study of 412 Labrador Retrievers found no difference in habitual activity between dogs with CCLR and those without CCLR [12]. Habitual activity was measured as general activity level, activity level at exercise, and ability to exercise. Similarly, there was no difference between groups with regard to the frequency of exercise or type of terrain on which dogs were most commonly exercised.

The sport of canine agility is growing in popularity worldwide and there has been a concomitant increase in interest in health management practices to maintain optimal athletic performance. This sport is especially physically demanding because it combines running and jumping, frequent abrupt turns at speed, navigation of elevated and angled frames or teeter-totters, and weaving between tightly spaced poles. Retrospective studies of agility dog injuries, based on handler reports, estimate that approximately one-third of agility dogs experience one or more injuries in their competitive career with one-third of those dogs having more than one injury. The most common anatomic sites reported to be injured are the shoulder, back, neck, and digits [13–21]. This study investigated the hypothesis that the risk of cruciate ligament rupture in dogs competing in agility would be increased in large breed dogs and in spayed female dogs, and decreased in dogs that were more physically active in agility training and competition, physical conditioning activities, and other dog sports.

## Methods

### Questionnaires

Internet-based questionnaires for dog owners were designed on a commercial internet survey site (Qualtrics, Provo, UT, www.qualtrics.com). The questionnaire for owners of dogs with CCLR included 61 items separated into 7 sections: introduction, pre-CCLR physical activities, description of the CCLR incident and its treatment, return to athletic activity after CCLR, dog signalment and physical characteristics, owner demographics, and consent to access performance records. The questionnaire for owners of dogs without CCLR included 26 items separated into 5 sections which were identical, except for the introduction, to the analogous sections in the questionnaire for CCLR dogs: introduction, physical activities, dog signalment and physical characteristics, owner demographics, and consent to access performance records. The two questionnaires are available as Supplementary Items 1 and 2. For this report, only data from the sections on pre-CCLR physical activities, dog signalment and physical characteristics, and owner demographics were analyzed.

Inclusion criteria for the CCLR group included a birth date between 1995 and 2014, participation in the sport of agility, and respondent age of at least 18 years. There were no requirements related to breed, sex, circumstances of the CCLR, or return to athletic activity after CCLR. Inclusion criteria for the control group dogs included a birth date between 1995 and 2014, participation in agility activities, no history of CCLR, and respondent age of at least 18 years.

A draft questionnaire was prepared and distributed to a small number of individuals who were actively engaged in dog agility. These individuals provided information related to required time for completion and clarity of the content. Based on the feedback received, minor modifications were made. The test responses from these individuals were deleted from the software before distribution of the final questionnaire.

The questionnaire for CCLR dogs was initiated on 9 September 2015 and remained open until 19 February 2016. The questionnaire for control dogs was initiated on 13 March 2016 and remained open until 27 March 2016. Invitations to participate in the surveys were distributed through social media sites and dog organizations that were relevant to agility enthusiasts. The questionnaire was accessed by clicking on a hyperlink in the message.

The Institutional Review Board of Washington State University determined this project satisfied the criteria for exempt research. The datasets generated and/or analyzed during the current study are not publicly available because they contain information that might breach respondent confidentiality. Anonymised subsets of data are available from the authors upon reasonable request.

### Data analysis

Responses to each question were summarised separately for CCLR and control dogs. Normality of data was assessed with a Shapiro-Wilk test. Descriptive statistics were calculated as median value with 25th and 75th percentiles for data that were not normally distributed. Categorical variables related to dog signalment and physical characteristics and specific physical activities were compared between CCLR and control dogs using Chi-square analysis with calculation of odds ratios and 95% confidence intervals (CI).

Continuous variables (age, height, weight, weight/height, body condition score, number of years involved in agility, and number of dogs the respondent had handled in agility) were compared using Mann-Whitney Rank Sum tests. Breed analysis was performed separately for each breed with 5 or more representatives in either the CCLR or control group. Odds ratios for each individual breed were calculated as compared to all other dogs. Corrections for multiple comparisons were calculated using the Benjamini-Hochberg method to control false discovery rates [22].

Separate multiple logistic regression analyses were performed to assess the relationship of agility-related activities of the dogs and non-agility physical activities including engagement in other dog sports and various physical conditioning activities with CCLR. Independent variables which were significant at *P* <  0.05 in univariate analysis were included in backward stepwise analysis to identify a subset of independent predictors significantly associated with the dependent variable of CCLR. These variables were included in calculation of the final multiple logistic regression models. All statistical analyses were performed using commercial statistical software (SigmaStat 4.0, Jandel Scientific, San Jose, CA) with significance determined at *P* <  0.05, unless specified otherwise.

## Results

### Questionnaire responses

A total of 677 respondents began the questionnaire for CCLR dogs; 411 responses were eliminated because the respondent failed to complete the questionnaire or because demographic data were incomplete or illogical. Three individuals submitted duplicate entries and responses were not consistent between the duplicates so all 6 of these responses were excluded from analysis (both entries for each dog removed). The final data set for CCLR dogs included 260 dogs. Of the 1492 individuals who accessed the control dog questionnaire, 486 respondents were eliminated because surveys were incomplete or had missing or illogical demographic data. The final data set for control dogs included 1006 dogs with no history of CCLR.

### Dog and handler demographics

There was no significant difference in agility experience of respondents based on comparison of number of years involved in agility and number of agility dogs handled. There was a significant difference between CCLR and control dogs in age, body weight, weight to height ratio, and body condition score (Table 1). In Chi-square analysis, there was a significant difference based on gender and neuter status with spayed female dogs at higher risk of CCLR (Table 2).

**Table 1:** Dog age and body size variables and handler agility experience for CCLR and control dogs

Variable	CCLR Median (25, 75%)	Control Median (25–75%)	***P***-value
Age	6 (4, 8)	7 (5, 10)	< 0.001
Dog height at withers (cm)	52 (44, 56)	51 (43, 56)	0.5
Dog weight (kg)	19 (14, 27)	18 (13, 23)	0.005
Dog weight/height ratio	0.38 (0.32, 0.46)	0.35 (0.29, 0.42)	< 0.001
Dog body condition score (1 to 5)^a^	3 (2, 3)	3 (2, 3)	0.008
Number of years handler has been involved in agility	12 (8, 16)	12 (7, 16)	0.2
Number of dogs handler has handled	3 (2, 5)	3 (2, 5)	0.2

Data shown are median values with 25th and 75th percentiles. *CCLR* cranial cruciate ligament rupture. ^a^ CCLR and control dogs had identical median values for dog body condition score but the Mann Whitney rank sum (MWRS) test detected a significant difference between the groups, with a higher mean body score for CCLR dogs (2.7) than for control dogs (2.6). The MWRS test compares mean ranks and not median values

**Table 2:** Chi-square analysis of dog sex and neuter status in CCLR and control dogs

Variable	CCLR Number (%)	Control Number (%)	Odds Ratio	95% CI	***P***-value	Chi-square
Male, intact	28 (10.8%)	149 (15.0%)	**Reference**	**Reference**	**Reference**	20.8 (*P* < 0.001)
Male, altered	79 (30.5%)	340 (34.2%)	1.2	0.7–2.0	0.4	
Female, intact	11 (4.2%)	102 (10.3%)	0.6	0.3–1.2	0.2	
Female, altered	141 (54.4%)	403 (40.5%)	1.9	1.2–2.9	0.008	

*CCLR* cranial cruciate ligament rupture, *CI* confidence interval

**Fig. 1:**
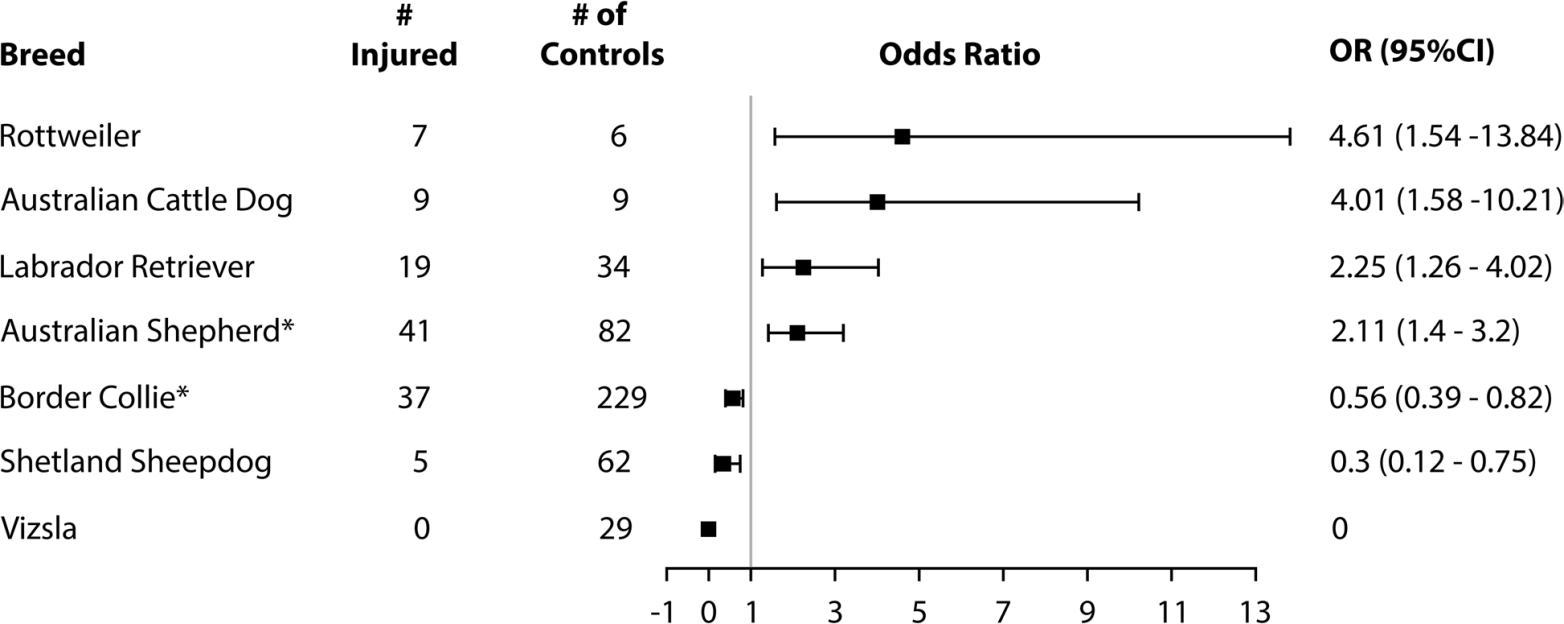
Odds ratio (OR) of cranial cruciate ligament rupture and corresponding 95% confidence interval (95% CI) in 118 dogs participating in agility compared to 451 control dogs. Based on Chi-square analysis, four breeds of dogs had increased OR for cranial cruciate ligament rupture and three breeds had decreased OR. *After a Benjamini-Hochberg correction for false discovery rates based on the number of breeds in the comparison, the increased odds ratio for Australian Shepherds and decreased odds ratio for Border Collies retained significance (*P* ≤ 0.05)

Breeds with significantly increased odds ratios for CCLR based on initial analysis included Australian Shepherd, Australian Cattle Dog, Labrador Retriever, and Rottweiler (Fig. 1). Breeds with decreased odds ratios included Border Collie, Shetland Sheepdog, and Vizsla. When corrected for false discovery rates only the Australian Shepherd and Border Collie breeds retained significance for increased and decreased odds of CCLR, respectively.

### Dog sport and conditioning activities

Odds ratios and 95% CI were calculated for physical activities of dogs including characterization of agility-specific activities (Table 3), involvement in other canine sports (Table 4), and weekly conditioning activities (Table 5). Frequency of engagement in conditioning activities was examined more closely to determine whether frequency of those activities impacted risk of CCLR (Table 6).

**Table 3:** Comparison of agility-specific activities of CCLR dogs prior to rupture and control dogs

Variable	CCLR Number (%)	Control Number (%)	Odds Ratio	95% CI	***P*** value
Train 1–7 days/week	241 (92.7%)	895 (89.0%)	**Reference**	0.4–1.1	0.1
Train < 1 day/week	19 (7.3%)	111 (11.0%)	0.6		
High level competition	161 (62.2%)	789 (79.5%)	**Reference**	1.8–3.2	< 0.001
Low to middle level competition	98 (37.8%)	204 (20.5%)	2.4		
< 10 competitions/year	117 (45.0%)	254 (25.2%)	1.4	1.1–1.8	0.02
> 10 competitions/year	143 (55.0%)	752 (74.8%)	**Reference**		
AKC competition	181 (69.6%)	684 (68.0%)	1.1	0.8–1.5	0.7
No AKC competition	79 (30.4%)	322 (32.0%)	**Reference**		
NADAC competition	90 (34.6%)	197 (19.6%)	2.2	1.6–2.9	< 0.001
No NADAC competition	170 (65.4%)	809 (80.4%)	**Reference**		
USDAA competition	87 (33.5%)	420 (41.7%)	0.7	0.5–0.9	0.02
No USDAA competition	173 (66.5%)	586 (58.3%)	**Reference**		
CPE competition	68 (26.2%)	231 (23.0%)	1.2	0.9–1.6	0.3
No CPE competition	192 (73.8%)	775 (77.0%)	**Reference**		
UKI competition	17 (6.5%)	194 (19.3%)	0.3	0.2–0.5	< 0.001
No UKI competition	243 (93.5%)	812 (80.7%)	**Reference**		
ASCA competition	41 (15.8%)	108 (10.7%)	1.6	1.1–2.3	0.033
No ASCA competition	219 (84.2%)	898 (89.3%)	**Reference**		
AAC competition	17 (6.5%)	150 (14.9%)	0.4	0.2–0.7	< 0.001
No AAC competition	243 (93.5%)	856 (85.1%)	**Reference**		

*CCLR* cranial cruciate ligament rupture, *CI* confidence interval, *AKC* American Kennel Club, *NADAC* North American Dog Agility Council, *USDAA* United States Dog Agility Association, *CPE* Canine Performance Events, *UKI* United Kingdom Agility International, *ASCA* Australian Shepherd Club of America, *AAC* Agility Association of Canada

**Table 4:** Participation in other canine sports by CCLR dogs prior to rupture and by control dogs

Variable	CCLR Number (%)	Control Number (%)	Odds Ratio	95% CI	***P***-value
Conformation	42 (16.2%)	229 (22.8%)	0.7	0.5–0.9	0.03
No conformation	218 (83.8%)	777 (77.2%)	**Reference**		
Flyball	31 (11.9%)	75 (7.5%)	1.7	1.1–2.6	0.03
No flyball	229 (88.1%)	931 (92.5%)	**Reference**		
Herding or stock dog	45 (17.3%)	227 (22.6%)	0.7	0.5–1.0	0.08
No herding or stock dog	215 (82.7%)	779 (77.4%)	**Reference**		
Obedience	108 (41.5%)	427 (42.4%)	1	0.7–1.3	0.8
No obedience	152 (58.5%)	579 (57.6%)	**Reference**		
Rally	96 (36.9%)	393 (39.1%)	0.9	0.7–1.2	0.6
No rally	164 (63.1%)	613 (60.9%)	**Reference**		
Disc dog	20 (7.7%)	101 (10.0%)	0.7	0.5–1.2	0.3
No disc dog	9 (3.5%)	905 (90.0%)	**Reference**		
Dock jumping	15 (5.8%)	110 (10.9%)	0.5	0.3–0.9	0.02
No dock jumping	245 (94.2%)	896 (89.1%)	**Reference**		
Lure coursing or racing	15 (5.8%)	117 (11.6%)	0.5	0.3–0.8	0.008
No lure coursing or racing	245 (94.2%)	889 (88.4%)	**Reference**		
Nosework	15 (5.8%)	155 (15.4%)	0.3	0.2–0.6	< 0.001
No nosework	245 (94.2%)	851 (84.6%)	**Reference**		
Barn hunt or earth dog	10 (3.8%)	143 (14.2%)	0.2	0.1–0.5	< 0.001
No barn hunt or earth dog	250 (96.2%)	863 (85.8%)	**Reference**		
Mushing	2 (0.8%)	30 (3.0%)	0.3	0.06–1.1	0.07
No mushing	258 (99.2%)	976 (97.0%)	**Reference**		
Protection	4 (1.5%)	19 (1.9%)	0.8	0.3–2.4	0.9
No protection	256 (98.5%)	987 (98.1%)	**Reference**		
Weight pull	1 (0.4%)	13 (1.3%)	0.3	0.04–2.3	0.4
No weight pull	259 (99.6%)	993 (98.7%)	**Reference**		
Involved in at least one other dog sport	196 (75.4%)	792 (78.7%)	0.8	0.6–1.1	0.3
Not involved in any other dog sports	64 (24.6%)	214 (21.3%)	**Reference**		

*CI* confidence interval

**Table 5:** Physical conditioning activities performed by CCLR dogs prior to rupture and by control dogs^a^

Variable	CCLR Number (%)	Control Number (%)	Odds Ratio	95% CI	***P*** value
Fetch games (e.g., ball or Frisbee)	171 (75.3%)	607 (80.1%)	0.8	0.5–1.1	0.1
No fetch games	56 (24.7%)	151 (19.9%)	**Reference**		
Swimming	57 (26.0%)	160 (23.7%)	1.1	0.8–1.6	0.5
No swimming	162 (74.0%)	515 (76.3%)	**Reference**		
Core strength, balance, stretching, and body awareness exercises	91 (38.9%)	479 (57.9%)	0.5	0.3–0.6	< 0.001
No core exercises	143 (61.1%)	348 (42.1%)	**Reference**		
Running and playing with other dogs	196 (83.4%)	727 (85.5%)	0.9	0.6–1.3	0.5
No running and playing with other dogs	39 (16.6%)	123 (14.5%)	**Reference**		
Short hikes or runs (< 30 min) on flat terrain	132 (59.2%)	256 (44.9%)	1.8	1.2–2.4	< 0.001
No short hikes or runs on flat terrain	91 (40.8%)	314 (55.1%)	**Reference**		
Long hikes or runs on flat terrain	78 (35.9%)	191 (39.1%)	0.9	0.6–1.2	0.5
No long hikes or runs (> 30 min) on flat terrain	139 (64.1%)	298 (60.9%)	**Reference**		
Short hikes or runs (< 30 min) on hilly terrain	100 (44.4%)	170 (30.5%)	1.8	1.3–2.5	< 0.001
No short hikes or runs on hilly terrain	125 (55.6%)	387 (69.5%)	**Reference**		
Long hikes or runs (> 30 min) on hilly terrain	74 (33.0%)	201 (30.4%)	1.1	0.8–1.6	0.5
No long hikes or runs on hilly terrain	150 (67.0%)	460 (69.6%)	**Reference**		
Short walks (< 30 min)	157 (74.8%)	606 (83.0%)	0.6	0.4–0.9	0.009
No short walks	53 (25.2%)	124 (17.0%)	**Reference**		
Long walks (> 30 min)	125 (58.1%)	468 (65.6%)	0.7	0.5–1.0	0.05
No long walks	90 (41.9%)	245 (34.4%)	**Reference**		

^a^Activities performed at least once weekly. *CCLR* cranial cruciate ligament rupture, *CI* confidence interval

**Table 6:** Association of frequency of specific conditioning activities with CCLI in agility dogs

Variable	Frequency	CCLI Number (%)	Control Number (%)	Odds Ratio	95% CI	***P***-value
Running and playing with other dogs	daily	156 (66.4%)	556 (65.4%)	0.7	0.5 - 1.1	0.2
	3-4 times per week	25 (10.6%)	105 (12.4%)	0.6	0.3 - 1.1	0.2
	1-2 times per week	15 (6.4%)	66 (6.8%)	0.6	0.3 - 1.2	0.2
	every other week	5 (2.1%)	34 (4.0%)	0.4	0.1 - 1.1	0.1
	less often	34 (14.5%)	89 (10.5%)	**Reference**		
Fetch games (e.g., ball or frisbee)	daily	92 (40.5%)	276 (36.4%)	0.8	0.5 - 1.2	0.4
	3-4 times per week	50 (22.0%)	193 (25.5%)	0.6	0.4 - 1.0	0.06
	1-2 times per week	29 (12.8%)	138 (18.2%)	0.5	0.3 - 0.9	0.02
	every other week	8 (3.5%)	34 (4.5%)	0.6	0.2 - 1.3	0.3
	less often	48 (21.1%)	117 (15.4%)	**Reference**		
Short walks (<30 min)	daily	74 (35.2%)	327 (44.8%)	0.6	0.4 - 0.9	0.02
	3-4 times per week	42 (20.0%)	160 (21.9%)	0.7	0.4 - 1.1	0.1
	1-2 times per week	41 (19.5%)	119 (16.3%)	0.9	0.5 - 1.5	0.7
	every other week	18 (8.6%)	36 (4.9%)	1.3	0.6 - 2.5	0.6
	less often	35 (16.7%)	88 (12.1%)	**Reference**		
Long walks (>30 min)	daily	27 (12.6%)	152 (21.3%)	0.4	0.3 - 0.7	0.002
	3-4 times per week	48 (22.3%)	162 (22.8%)	0.7	0.5 - 1.1	0.2
	1-2 times per week	50 (23.3%)	154 (21.6%)	0.8	0.5 - 1.2	0.4
	every other week	23 (10.7%)	78 (11.0%)	0.7	0.4 - 1.3	0.3
	less often	67 (31.2%)	166 (23.3%)	**Reference**		
Short hikes or runs (<30 min), flat terrain	daily	43 (19.3%)	52 (9.1%)	2.6	1.6 - 4.3	<0.001
	3-4 times per week	46 (20.6%)	89 (15.6%)	1.6	1.1 - 2.6	0.03
	1-2 times per week	43 (19.0%)	115 (20.2%)	1.2	0.8 - 1.8	0.5
	every other week	16 (7.2%)	75 (13.2%)	0.7	0.4 - 1.2	0.3
	less often	75 (33.6%)	239 (41.9%)	**Reference**		
Short hikes or runs (<30 min), rough/hilly terrain	daily	28 (12.4%)	29 (5.2%)	2.9	1.7 - 5.1	<0.001
	3-4 times per week	27 (12.0%)	54 (9.7%)	1.5	0.9 - 2.5	0.2
	1-2 times per week	45 (20.0%)	87 (15.6%)	1.5	1.0 - 2.4	0.05
	every other week	16 (7.1%)	60 (10.8%)	0.8	0.4 - 1.4	0.6
	less often	109 (48.4%)	327 (58.7%)	**Reference**		
Long hikes or runs (>30 min), flat terrain	daily	12 (5.5%)	34 (5.8%)	1.0	0.5 - 1.9	0.9
	3-4 times per week	22 (10.1%)	68 (11.5%)	0.9	0.5 - 1.5	0.8
	1-2 times per week	44 (20.3%)	89 (15.1%)	1.4	0.9 - 2.1	0.2
	every other week	20 (9.2%)	71 (12.1%)	0.8	0.5 - 1.3	0.8
	less often	119 (54.8%)	327 (55.5%)	**Reference**		
Long hikes or runs (>30 min), rough/hilly terrain	daily	14 (6.3%)	21 (3.2%)	2.0	1.0 - 4.0	0.09
	3-4 times per week	18 (8.0%)	56 (8.5%)	1.0	0.5 - 1.7	1.0
	1-2 times per week	42 (18.8%)	124 (18.8%)	1	0.7 - 1.5	0.9
	every other week	16 (7.1%)	64 (9.7%)	0.7	0.4 - 1.3	0.4
	less often	134 (59.8%)	396 (59.9%)	**Reference**		
Swimming	daily	6 (2.7%)	22 (3.3%)	0.9	0.3 - 2.2	0.9
	3-4 times per week	20 (9.1%)	52 (7.7%)	1.2	0.7 - 2.1	0.6
	1-2 times per week	31 (14.2%)	86 (12.7%)	1.1	0.7 - 1.8	0.6
	every other week	25 (11.4%)	79 (11.7%)	1.0	0.6 - 1.6	0.9
	less often	137 (62.6%)	436 (64.6%)	**Reference**		
Core strength, balance, stretching, and body awareness exercises (e.g. wobble board, FitPaws, trick training)	daily	10 (4.3%)	46 (5.6%)	0.5	0.2 - 1.0	0.05
	3-4 times per week	28 (12.0%)	172 (20.8%)	0.4	0.2 - 0.5	<0.001
	1-2 times per week	53 (22.6%)	261 (31.6%)	0.4	0.3 - 0.6	<0.001
	every other week	22 (9.4%)	88 (10.6%)	0.5	0.3 - 0.9	0.02
	less often	121 (51.7%)	260 (31.4%)	**Reference**		

*CCLI* cranial cruciate ligament injury, *CI* confidence interval.

Dogs in the CCLR group were significantly more likely to compete in agility at a lower level (e.g. novice or intermediate), compete fewer than 10 times per year, and compete in events sponsored by North American Dog Agility Council (NADAC) or the Australian Shepherd Club of America (ASCA). These dogs were less likely to compete in events sponsored by United States Dog Agility Association (USDAA), United Kingdom Agility International (UKI), and Agility Association of Canada (AAC). These variables which were significant in univariate analysis were included in backward stepwise analysis with the dependent variable of CCLR and competition level as a forced variable. Participation in AAC events was not included in this analysis because of the low numbers of individuals indicating involvement. Variables which retained significance were included in the final logistic regression model shown in Table 7. This model was judged to be a good fit with a Hosmer-Lemeshow statistic of 6.728 (*P* = 0.57) and likelihood ratio test statistic of 91.7 (*P* <  0.001).

**Table 7:** Final multivariable logistic regression model of agility-associated activities as risk factors for CCLR

Variable	Coefficient	Standard Error	Wald Statistic	***P*** Value	Odds Ratio	95% CI
Engaged in NADAC competitions	0.70	0.20	20.7	< 0.001	2.0	1.5–8.9
Not engaged in NADAC competitions					**Reference**	
Engaged in UKI competitions	−1.10	0.30	16.4	< 0.001	0.3	0.2–0.6
Not engaged in UKI competitions					**Reference**	
Competition at lower levels (Novice, Intermediate, Open)	0.60	0.20	12.0	< 0.001	1.8	1.3–2.5
Competition at higher levels (Master’s Excellent, Elite)					**Reference**	
Fewer than 10 competitions/year	0.60	0.20	12.3	< 0.001	1.8	1.3–2.4
More than 10 competitions/year					**Reference**	

*CCLR* cranial cruciate ligament rupture, *NADAC* North American Dog Agility Council, *UKI* United Kingdom Agility International, *CI* confidence interval

Dogs with CCLR were more likely to engage in the sports of flyball and less likely to engage in conformation, dock jumping, lure coursing or racing, nosework, and barn hunt or earth dog activities. Dogs with CCLR were more likely to engage in short hikes or runs on flat or hilly terrain on at least a weekly basis. These dogs were less likely to engage in weekly or more frequent exercises related to core strength and balance or go on weekly short or long walks. The conditioning and alternative sport variables which were significant in this univariate analysis were included in backward stepwise analysis with the dependent variable of CCLR. Variables which retained significance were included in the final logistic regression model shown in Table 8. This model was judged to have a good fit with a Hosmer-Lemeshow statistic of 9.77 (*P* = 0.28) and likelihood test statistic of 85.7 (*P* <  0.001).

**Table 8:** Final multivariable logistic regression model of other activities as risk factors for CCLR

Variable	Coefficient	Standard Error	Wald Statistic	***P*** Value	Odds Ratio	95% CI
Barn hunt or earth dog activities	−1.1	0.4	8.0	0.005	0.3	0.2–0.7
No barn hunt or earth dog activities					**Reference**	
Nosework activities	−1.0	0.3	8.9	0.003	0.4	0.2–0.7
No nosework activities					**Reference**	
Dock diving activities	−0.8	0.3	6.4	0.01	0.4	0.2–0.8
No dock diving activities					**Reference**	
Flyball activities	0.7	0.3	5.6	0.02	2.0	1.1–3.4
No flyball activities					**Reference**	
Lure coursing or racing activities	−0.7	0.3	4.2	0.04	0.5	0.3–1.0
No lure coursing or racing activities					**Reference**	
Short (< 30 min) hikes or runs on hilly terrain	0.6	0.2	9.5	0.002	1.8	1.3–2.7
No short (< 30 min) hikes or runs on hilly terrain					**Reference**	
Short (< 30 min) hikes or runs on flat terrain	0.6	0.2	8.6	0.003	1.7	1.2–2.5
No short (< 30 min) hikes or runs on flat terrain					**Reference**	
Core strength, balance, stretching, and body awareness exercises	−0.5	0.2	6.7	0.009	0.6	0.4–0.9
No core exercises					**Reference**	

*CCLR* cranial cruciate ligament rupture, *CI* confidence interval

## Discussion

This study reports on dog characteristics and physical activities associated with risk of CCLR in a large group of agility dogs based on information provided by owners. Findings were consistent with previous reports that spayed female dogs of large and giant breeds are at increased risk of CCLR. Competing at higher levels of agility, competing more frequently, and competing in more technically challenging events was associated with a decreased incidence of CCLR. Engagement in specific conditioning activities or other canine sports was associated with either increased risk of CCLR (flyball, daily hikes or runs) or decreased risk of CCLR (core strengthening and balance exercises, barn hunt or earth dog, nosework, lure coursing or racing).

Cranial cruciate ligament disease in dogs is characterized by degeneration of the extracellular matrix of the ligament, leading to eventual ligament rupture [23]. Acute rupture may also occur as a result of direct trauma to a healthy stifle joint. It is not possible to ascertain whether the dogs in this report had typical degenerative lesions which led to rupture, acute trauma that occurred during sports participation, or a combination of these two processes which led to eventual CCLR. Considering that the demographic risk factors identified are similar to risk factors previously reported for CCLR [23], it is likely that typical CCL disease occurred in most affected dogs. The questionnaire used for this study referred to cruciate ligament “tears” because this was terminology that dog owners were likely to understand. In this manuscript, consistent with the terminology used in some current literature [23], the term cranial cruciate ligament rupture was used regardless of whether there was a complete or partial ligament rupture and without any attempt to discern the etiopathogenesis of rupture in individual dogs.

This study compared dogs with CCLR to a large group of control dogs which had no history of CCLR. The type or characteristics of CCLR (e.g. partial rupture, complete rupture, meniscal injury) was not explored in this analysis but might influence results and provide more nuanced information about agility dog injuries. The control group was representative of the broad cross-section of types of dogs which compete in agility in the United States. Approximately 90% of respondents indicated that they competed in events hosted by agility organizations within the United States. The distribution of breeds within the control group was consistent with previous reports. Overall, almost one-quarter of control dogs were Border Collies. It is possible that some of the dogs in the control group experienced CCLR later in their athletic career, after the survey information was submitted, but the numbers of such dogs are likely small. Levy, et al. reported that approximately 10% of agility dogs experience “stifle injury” during their career [14]. The signalment and characteristics of dogs at increased risk for CCLR in this study, however, are very similar to those reported in general canine population, suggesting that the control data set was appropriately representative.

### Signalment and dog characteristics

The signalment factors associated with risk of CCLR in this study were generally consistent with previous reports of increased risk of CCLR in spayed female dogs, large breed dogs, and dogs with increased body weight [1, 6, 7, 11, 24–29]. CCLR dogs had a lower median age than control dogs. In previous reports of CCLR, risk of rupture appeared to increase in older dogs [6, 11] although age at diagnosis tends to be lower with increasing size of the dog [11]. One report suggests that risk for middle aged dogs (4 to 7 years) may be slightly higher than for dogs > 7 years of age [1]. The lower age of CCLR dogs in this study is consistent with the observation of increased risk in dogs competing at lower levels (e.g. novice, open, or intermediate levels) in agility.

The increased risk of CCLR in Labrador Retrievers and Rottweilers has been reported previously [1, 4, 6–8, 11]. Breed analysis varied slightly from previous studies, however, in the recognition of increased risk of CCLR in Australian Shepherds and Australian Cattle Dogs. Risk of “joint disorder” in specific breeds, defined as CCLR and hip dysplasia, was assessed by Hart, et al. in relation to neuter status and age at time of neutering [26]. There was a 3–4% rate of joint disorders including CCLR in intact male and female Australian Shepherds with no evidence of increased risk with neutering at any age. This was similar to Border Collies, which had a 2–3% risk of joint disease with no increased risk with neutering. In contrast, Labrador Retrievers had a 6% risk in intact males and females and an 11–13% risk for neutered animals [26]. It is surprising, therefore, that Australian Shepherds participating in agility had such a strong statistical risk for CCLR in this study.

The increased risk associated with Australian Shepherds could be the result of selection and response bias due, in part, to biased survey distribution which attracted an unusual number of Australian Shepherd enthusiasts. This bias seems less likely given the relatively large number of dogs included in the control group. If the risk for CCLR is truly increased for Australian Shepherds, it may be the result of a specific factor that influences this breed when performing in the sport of agility. Body conformation, increased weight, or greater weight to height ratio for these dogs may increase risk of rupture with sporting activities. This was not apparent in exploratory analysis comparing weight to height ratios of Australian Shepherds in the CCLR group to those in the control group (data not shown). Similarly, there was no difference between Australian Shepherd dogs and all other breeds in the proportion that were spayed females but there were very few intact female Australian Shepherds in either the CCLR group (*n* = 2) or the control group (*n* = 1).

In the United States, Australian Shepherds typically have surgically docked or naturally bobbed tails and the Australian Shepherd Club of America includes in its breed standard that an identifying characteristic of the breed is “his natural or docked bobtail” [30]. In running quadrupeds, the tail provides counterbalance and enables turning at higher speed [31–33]. Tail motion is likely important for counterbalance and muscle function in jumping, negotiating balance obstacles like the dog walk, and performance of weave poles at high speed. The absence of a tail may result in diminished balance or change in motion or limb loading characteristics that predispose to CCLR in dogs participating in agility. This cannot be confirmed with available data because owners were not asked whether their dogs had normal or docked tails. Additional study to confirm the increased risk of Australian Shepherds and identify reasons for that increased risk is recommended.

### Agility-related factors

The agility-related factors associated with increased odds ratio for CCLR included competing at a lower level (e.g. novice, intermediate, open), competing in fewer events per year, and competing in NADAC-sponsored events. There was a decreased odds ratio for dogs competing in UKI-sponsored events. Together, the final logistic regression model suggests that more experienced dogs competing more frequently in more technically challenging types of competition have a lower risk of CCLR. These results seem counter-intuitive, but in combination with the data related to other physical and conditioning activities of dogs as discussed below, may indicate that the increased physical fitness required to compete frequently at higher levels in technically challenging venues may offset or decrease risk associated with the more physically demanding activities themselves. Dogs that are more physically fit may experience less fatigue during competition. Fatigue has been postulated to increase the risk of cruciate ligament rupture in human athletes [34, 35].

The difference in odds ratios associated with competition in different agility venues was unexpected. In NADAC competitions, an emphasis is placed on asking the dog to work at a greater distance from the handler. NADAC agility is characterised by generally lower jump height options for many dogs, greater distances between obstacles (range of 18 to 24 ft), lesser emphasis on tight turns, and more non-jumping obstacles such as hoops, barrels, and tunnels [36]. Because of these considerations, ground speed in yards/second for elite dogs may be higher than for elite dogs competing in other agility venues and it is possible that increased risk for CCLR is related to these higher speeds. This seems unlikely, however, given that the increased odds ratios are observed with less experienced dogs which are not competing at an elite level. UKI agility events are characterised by generally higher jump heights, more tightly spaced obstacles (minimum distance of 12 ft), tighter turns at speed, and options for back side approaches to jumps, threadles, and similarly athletically challenging course elements [37]. The decreased odds ratio associated with UKI competition may be the consequence of a greater fitness level required to compete at elite levels in this venue. Potential differences in ground surfaces among agility venues could influence the risk of CCLR. For running greyhounds, ground compliance significantly impacts the forces acting on the pelvic limbs [38] and ground surfaces while racing influence the risk of injury [39].

### Conditioning activities

The relationship between CCLR and physical conditioning activities explored in this work is complicated. Some activities such as fetch games with a ball or disc and swimming had no discernible relationship to CCLR risk. Interpretation of data related to walking, hiking, and running activities is more nuanced. It appears that short or long walks such as occur on a casual leash walk may be associated with decreased risk of CCLR if done daily but minimal positive or negative effect when done on a less frequent basis. In contrast, short or long hikes or runs are likely detrimental if done daily regardless of whether terrain is smooth or rough. The increase in risk of CCLR associated with hikes and runs relative to walks could be a direct consequence of repeated microtrauma from forces acting on the pelvic limbs when running and hiking compared to walking or could be the indirect consequence of fatigue from more strenuous daily activity with decrease in the protective mechanisms of the stifle joint [35]. The latter theory would be consistent with the decreased risk that was observed in dogs engaging in regular core strength, balance, and body-awareness exercises.

The protective effect of regular core strength and balance exercises occurred regardless of frequency and retained significance in the multivariable model. Factors related to core stability predict risk of cruciate ligament injuries with high sensitivity and moderate specificity in female human athletes but not in male athletes [40]. Injury prevention programs that incorporate core strengthening exercises might be beneficial for agility dogs. Exercises to increase core strength are commonly included in conditioning programs that are beneficial in prevention of anterior cruciate rupture in human athletes [41–43].

### Participation in other canine sports

Most of the dogs in both the control and CCLR groups participated in other canine sports in addition to agility (75.4 and 78.7%, respectively; *P* = 0.3). Participation in specific sports, however, had a variable effect. Obedience and rally were the most frequently cited canine sports in which respondents were involved, but participation in these sports was not associated with increased or decreased risk of CCLR. It was surprising that participation in less strenuous sports such as barn hunt/earth dog and nosework was associated with decreased odds of CCLR. These sports require very different types of physical exertion as compared to canine agility. Barn hunt and earth dog competitors are required to climb over obstacles and crawl through small spaces; these activities may be associated with increased core strength and stability. Both barn hunt and nosework require a high degree of communication and teamwork between handler and dog. Dogs are trained to present behavioral cues to the handler and the handler must recognize these sometimes-subtle cues and respond appropriately. Participation in these sports may improve or enhance the human-animal bond in a way that enhances communication in agility activities, with resultant decreased CCLR in dogs. Other sports associated with decreased risk of CCLR in agility dogs included dock diving and lure coursing or racing. These activities require a generally high level of athleticism and fitness but do not require rapid changes in direction or movement over rough terrain. Participation in these sports might increase overall fitness level without increasing risk of CCLR.

The only canine sport associated with increased risk of CCLR in agility dogs was flyball. This sport requires that dogs race from a start line over a series of 4 hurdles to a spring-loaded box that releases a tennis ball. The dog catches the tennis ball and pivots, banking off the release box, to race back to the start line with the ball. Injuries in flyball dogs are common with approximately 39% of dogs incurring at least one injury in a report by Montalbano, et al. [44] and 34% in a report by Pinto, et al. [45] The rapid turns, speed, and jumping may increase risk of CCLR but more study is needed to identify specific risk factors associated with flyball participation.

Veterinary health data obtained directly from owners through internet-based questionnaires rather than from veterinary medical records are subject to possible sampling, confirmation, and recall bias [46]. There are few or no veterinary facilities, however, that examine and treat sufficient numbers of dogs actively engaged in agility to produce studies of adequate power to draw reliable conclusions about specific types of injuries based solely on review of medical records. As a result, internet-based questionnaires for assessing the health of agility dogs are being used with increasing frequency. Owners of agility dogs are often quite observant and diligent in providing care for their dogs, but there is minimal information about the potential lack of accuracy of data obtained in this manner [18]. This study had no independent confirmation of diagnosis of CCLR; dogs were classified as CCLR or control (no CCLR) based solely on the information provided by the owner. Approximately 78% of dogs in the CCLR group were treated surgically and a diagnosis was likely to be confirmed at that time. The precise diagnosis for the remaining 22% of CCLR dogs, for which no surgery was performed and there was no review of medical records, cannot be confirmed. The results of this study should, therefore, be interpreted with caution and careful consideration of the potential for distribution, respondent, and recall bias.

This study provides intriguing new information about possible risk factors for CCLR in agility dogs. This is the first report that has statistically linked other physical activities to increased or decreased risk of CCLR in agility dogs. Additional study is needed to better define the nature of the observed associations. The information in this report might be used to assist with the design of studies to assess injury prevention programs that include core strength and balance exercises. Agility organizations should prioritize and encourage research with the primary goal of improving participant health and safety. A previous recommendation to establish a comprehensive injury surveillance system to “provide a foundation for evidence-based decision making with regard to health and safety issues” within the sport of agility should be reviewed and considered [15].

## Supplementary Information

**Additional file 1.**

**Additional file 2.**

## Data Availability

The datasets used and/or analyzed during the current study are not currently available because they contain information that might breach respondent confidentiality but are available from the corresponding author on reasonable request.
