# Supplementary material for: Risk factors for cranial cruciate ligament rupture in dogs participating in canine agility
Source: BMC Vet Res. 2022 Jan 15;18:39. doi: 10.1186/s12917-022-03146-2 (PMC8760802; doi:10.1186/s12917-022-03146-2)
Supplement: Supplementary file 1 — Additional file 1. [file 12917_2022_3146_MOESM1_ESM.pdf]

Introduction

Purpose: Injuries to the cranial cruciate ligament in the knee (stifle) of agility dogs are common. The primary purpose of this study is to evaluate the return to performance in agility dogs after these injuries.

Eligibility: Any dog born between 1995-2014 that has competed in agility and has suffered a knee ligament injury is eligible to participate. All breeds/mixes and sexes are eligible. It is **not** required that the injury occurred during agility and it is **not** required that the dog returned to agility after the injury. However, the dog should have participated in agility prior to diagnosis and have completed their recovery. Owners completing this survey must be at least 18 years old.

Participation: Participation in this study is completely voluntary. No details about yourself or your dogs will be collected from this survey beyond what you choose to provide in your answers. You may quit the survey at any time and your answers to this survey will not be saved if you quit. Full participating in the study will require consent to access your dog's agility performance records and permission to contact your veterinarian with a follow-up survey regarding your dog's treatment.

Estimated Time: This survey takes approximately 20-30 minutes to complete. There will be detailed questions about your dog's training, performance, injury, and recovery. You can prepare by gathering information about your dog's agility history and veterinary information about their injury.

Who: The research team for this project includes Ms. Katherine Martucci (veterinary student at Washington State University), Dr. Debra Sellon (Professor of Equine Medicine at Washington State University), Dr. Denis Marcellin-Little (Professor of Small Animal Orthopedic Surgery at North Carolina State University), Dr. Michelle Powers (Small Animal Surgeon at Massachusetts Veterinary Referral Hospital), Dr. Kim Cullen (Research Coordinator at Canines in Motion), and Sarah Fernandezlopez.

Ethics: This study was deemed exempt from review by the Institutional Review Board (IRB) at Washington State University.

If you have any questions, comments, or concerns, please contact Katherine Martucci at kmartucci@vetmed.wsu.edu.

What is the name of the dog for which you are submitting information?

If you have multiple dogs eligible for this study please submit one survey for each of them separately.

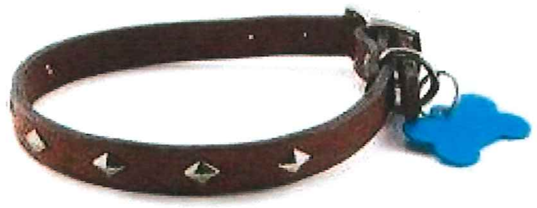

Routine pre-injury

We will start by asking some questions about ${q://QID1/ChoiceTextEntryValue}'s routine during the year **prior to his/her knee injury.** Please answer them to the best of your ability. You will later be asked similar questions about ${q://QID1/ChoiceTextEntryValue}'s routine after the injury.

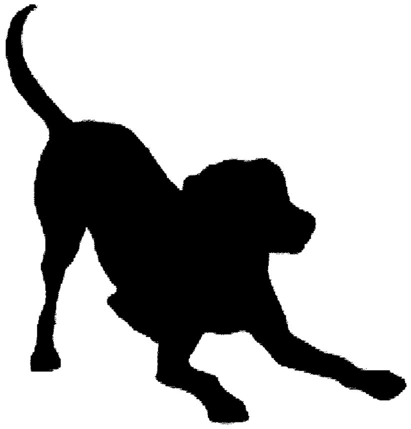

In which agilty venues did ${q://QID1/ChoiceTextEntryValue} compete prior to injury? Select all that apply.

AAC (Agility Association of Canada)

AKC (American Kennel Club)

ASCA (Australian Shepherd Club of America)

CPE (Canine Performance Events)

DOCNA (Dogs on Course in North America)

NADAC (North American Dog Agility Council)

UKC (United Kennel Club)

UKI (UK Agility International)

USDAA (United States Dog Agility Association)

International competition outside of North America

Other (please specify)

What is the highest level of agility that ${q://QID1/ChoiceTextEntryValue} had achieved in any venue prior to injury?

Training only - my dog has not competed in agility trials

Starters/Novice/Beginner

Advanced/Open/Intermediate

Masters/Elite/Excellent

International

Other - please specify

On average, how many competitive agility events did ${q://QID1/ChoiceTextEntryValue} participate in annually prior to injury?

1-5

6-10

11-15

16-20

More than 20

On average, how frequently did ${q://QID1/ChoiceTextEntryValue} train in agility prior to injury?

Daily

3-6 times per week

1-2 times/week

Less than once per week

Did ${q://QID1/ChoiceTextEntryValue} participate (either training or competing) in any of the following sports prior to injury? Please select all that apply.

Barn Hunt or Earth Dog

Conformation

Disc Dog

Dock Jumping

Flyball

Herding or Stock Dog

Hunting, Hunt Tests, or Field Trials

Lure Coursing or Racing

Mushing

Nosework

Obedience

Protection (e.g. Schutzhund, Ring Sport)

Rally

Weight Pull

Other, please specify

${q://QID1/ChoiceTextEntryValue} did not participate in any other sports

Did you do any weekly conditioning with ${q://QID1/ChoiceTextEntryValue} separate from sport training prior to injury? Please indicate the frequency with which ${q://QID1/ChoiceTextEntryValue} participated in each of the following activities by grouping the activities into the categories below.

Click on a conditioning item in the left column and drag/drop it into the appropriate frequency box on the right.

Items

Core strength, balance, stretching, and body awareness exercises (e.g., wobble board, FitPaws, trick training)

10 / 10

Daily

3-4 times per week

1-2 times per week

Every other week

Monthly

Rarely

Never

If desired, use this space to further describe any regular conditioning activities you did with ${q://QID1/ChoiceTextEntryValue} separate from sport training. (optional)

The injury, diagnosis and treatment

The next section will ask questions regarding the specifics of ${q://QID1/ChoiceTextEntryValue}'s injury and diagnosis.

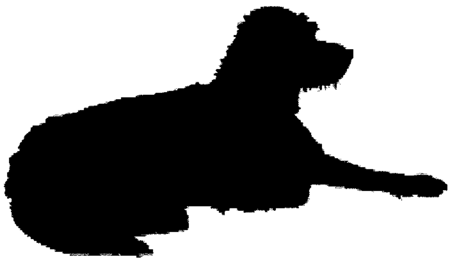

Which knee was injured?

Left

Right

${q://QID1/ChoiceTextEntryValue} has injured both knees

You indicated that ${q://QID1/ChoiceTextEntryValue} has injured both knees. Please respond to the remainder of the survey in respect to your dog's **first knee injury**. You will not be asked about the second injury specifically beyond this point.
${q://QID1/ChoiceTextEntryValue}'s **first knee injury** involved the:

Left limb

Right limb

Approximately when did ${q://QID1/ChoiceTextEntryValue}'s injury occur? Enter date in the format mm/dd/yyyy

	Month	Day	Year
Injury date			

How did ${q://QID1/ChoiceTextEntryValue}'s knee injury occur?

While free running or playing

While participating in agility (training or competition)

While participating in a different sport (please specify)

Other (please describe)

Not sure

What type of ground surface was ${q://QID1/ChoiceTextEntryValue} on when the injury occurred?

Dirt (dry)

Mud and/or water (e.g., ponds, lakes)

Sand

Grass (dry)

Grass (wet)

Artificial turf

Rubber mats

Carpet

Hard slick floor (e.g., wood, tile, linoleum)

Unsure

Other (please specify)

After noticing a problem, approximately how long was it before you took ${q://QID1/ChoiceTextEntryValue} to a veterinarian for evaluation of the injury?

The problem was first noticed by the veterinarian

Less than 72 hours

4-6 days

1-2 weeks

3-4 weeks

More than a month

Not sure

Who primarily diagnosed and treated ${q://QID1/ChoiceTextEntryValue}'s injury?

${q://QID1/ChoiceTextEntryValue}'s primary care veterinarian

A different primary care veterinarian that had not seen ${q://QID1/ChoiceTextEntryValue} previously

Referral or veterinary specialist

Other (please specify)

${q://QID1/ChoiceTextEntryValue} was diagnosed with a....

Partial cruciate tear

Complete cruciate tear

Neither

Now you will move on to questions about ${q://QID1/ChoiceTextEntryValue}'s treatment and recovery process.

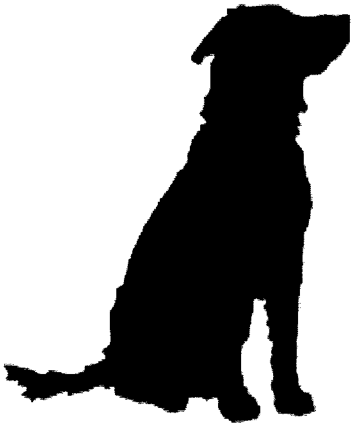

Was ${q://QID1/ChoiceTextEntryValue}'s injury treated surgically?

Yes

No

What type of surgery did ${q://QID1/ChoiceTextEntryValue} recieve?

Extra-capsular stabilization (aka: "ex-cap suture", "lateral suture", or "fishing line")

Tightrope stabilization

Tibial plateau leveling osteotomy (TPLO)

Tibial tuberosity advancement (TTA)

Intra-capsular stabilization (grafting)

Other

Not sure

What as the date of ${q://QID1/ChoiceTextEntryValue}'s knee surgery? Enter date in the format mm/dd/yyyy

	Month	Day	Year
Surgery date			

Did your veterinarian offer to include stem cell therapy or other regenerative therapy (e.g., platelet rich plasma) as part of ${q://QID1/ChoiceTextEntryValue}'s treatment plan?

Yes

No

Unsure

Did you include stem cell therapy or other regenerative therapy (e.g. platelet rich plasma) as part of ${q://QID1/ChoiceTextEntryValue}'s treatment plan?

Yes

No

Did you receive written rehabilitation instructions from the treating veterinarian for ${q://QID1/ChoiceTextEntryValue}'s recovery?

Yes

No

How closely do you feel you followed the veterinarian-provided rehabilitation instructions?

Very closely

Somewhat closely

Somewhat loosely

Not at all

Did you follow **any rehabilitation protocol** to assist with ${q://QID1/ChoiceTextEntryValue}'s recovery? (Whether or not the protocol was provided by the treating veterinarian)

Yes

No

With the agreed-upon treatment plan, what was the veterinarian's agility prognosis for ${q://QID1/ChoiceTextEntryValue}?

Full recovery and return to agility expected

Return to agility possible, but with some restrictions

Limited recovery with return to agility unlikely

No return to agility expected

Unspecified

What types of rehabilitation techniques were utilized during ${q://QID1/ChoiceTextEntryValue}'s recovery?

Click and drag each item on the left into the appropriate box on the right. Additionally, you can rank the therapies from most frequently used to least frequently used within each box by dragging/dropping to adjust.

Items

Knee brace

16 / 16

Therapies USED during ${q://QID1/ChoiceTextEntryValue}'s recovery (ranked from most to least frequently applied):

Therapies NOT used during ${q://QID1/ChoiceTextEntryValue}'s recovery:

Routine post-injury

Questions in the following section refer to ${q://QID1/ChoiceTextEntryValue}'s routine **at its peak after the injury treatment**. In other words, if considerable time has passed since ${q://QID1/ChoiceTextEntryValue}'s injury and treatment, please answer the following questions in relation to ${q://QID1/ChoiceTextEntryValue}'s routine and performance before any unrelated activity reductions due to age.

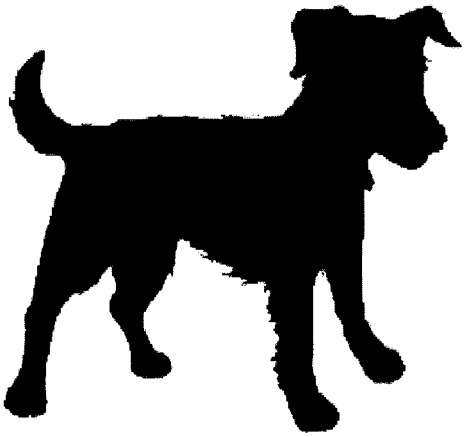

Approximately how long after injury (if non-surgically treated) or surgery (if applicable) did ${q://QID1/ChoiceTextEntryValue} recover to his/her full potential?

Drag the slider to the appropriate number of months. If longer than two years, check the box to the right.

	Months													Not Applicable
	0	2	4	6	8	10	12	14	16	18	20	22	24	
Months														☐

Did ${q://QID1/ChoiceTextEntryValue} return to agility?

Yes, but only training (never competition)

Yes, ${q://QID1/ChoiceTextEntryValue} returned to both training and competition

Not sure yet - ${q://QID1/ChoiceTextEntryValue} may return to agility in the future

No, ${q://QID1/ChoiceTextEntryValue} never returned to agility

Has ${q://QID1/ChoiceTextEntryValue} participated (either training or competing) in any of the following sports after injury? Please select all that apply.

Barn Hunt or Earth Dog

Conformation

Disc Dog

Dock Jumping

Flyball

Herding or Stock Dog

Hunting, Hunt Tests, or Field Trials

Lure Coursing or Racing

Mushing

Nosework

Obedience

Protection (e.g. Schutzhund, Ring Sport)

Rally

Weight Pull

Other, please specify

${q://QID1/ChoiceTextEntryValue} does not participate in any other sports

Since the injury and recovery, do you do any weekly conditioning with ${q://QID1/ChoiceTextEntryValue} separate from sport training? Please indicate the frequency with which ${q://QID1/ChoiceTextEntryValue} participates in each of the following activities by grouping the activities into the categories below.

Click on a conditioning item in the left column and drag/drop it into the appropriate frequency box on the right.

Items

Core strength, balance, stretching, and body awareness exercises (e.g., wobble board, FitPaws, trick training)

10 / 10

Daily	3-4 times per week

1-2 times per week	Every other week

Monthly	Rarely

Never

If desired, use this space to further describe any regular conditioning activities you have done with ${q://QID1/ChoiceTextEntryValue} separate from sport training since recovery. (optional)

Dog did not return to agility

You indicated that ${q://QID1/ChoiceTextEntryValue} did not return to agility competition. Please provide additional details about your answer.

Why did ${q://QID1/ChoiceTextEntryValue} not return to agility competition? Please select all that apply.

${q://QID1/ChoiceTextEntryValue} was/is physically incapable of competing in agility due to the knee injury

Recovery is still in progress but ${q://QID1/ChoiceTextEntryValue} may return to agility in the future

Retired from competition for reasons unrelated to the knee injury

Retired from competition for reasons unrelated to agility

Prefer not to say

Other, please specify

If you would like to provide additional details about why ${q://QID1/ChoiceTextEntryValue} did not return to agility competition please do so here:

Agility routine post-injury

You indicated that ${q://QID1/ChoiceTextEntryValue} returned to agility competition. Please provide additional details below.

Approximately how long after surgery (if applicable) or injury (if non-surgically treated) did ${q://QID1/ChoiceTextEntryValue} return to **full agility training**?

Drag the slider to the appropriate number of months. If longer than two years, check the box to the right.

	Months													Not Applicable
	0	2	4	6	8	10	12	14	16	18	20	22	24	
Months														☐

On average, how frequently did ${q://QID1/ChoiceTextEntryValue} **train in agility** after recovery?

Daily

3-6 times per week

1-2 times/week

Less than once per week

Did you make any changes to ${q://QID1/ChoiceTextEntryValue}'s agility performance criteria as a result of the cruciate injury and/or recovery?

Yes

No

What performance criteria did you change for ${q://QID1/ChoiceTextEntryValue} as a result of the injury and/or recovery? Please select all that apply

Lower jump height

Different contact performance

Avoid specific obstacles

Compete in a different program (e.g., Championship/Performance, Regular/Preferred)

Compete in different venues

Other (please describe)

What was the approximate date that ${q://QID1/ChoiceTextEntryValue} returned to agility competition? Enter date in the format mm/yyyy

	Month	Year
Date of first trial after recovery		

On average, how many **competitive agility events** did/does ${q://QID1/ChoiceTextEntryValue} participate in annually since recovery?

1-5

6-10

11-15

16-20

More than 20

In which agilty venues has ${q://QID1/ChoiceTextEntryValue} competed in after recovery? Select all that apply.

AAC (Agility Association of Canada)

AKC (American Kennel Club)

ASCA (Australian Shepherd Club of America)

CPE (Canine Performance Events)

DOCNA (Dogs on Course in North America)

NADAC (North American Dog Agility Council)

UKC (United Kennel Club)

UKI (UK Agility International)

USDAA (United States Dog Agility Association)

International competition outside of North America

Other (please specify)

What is the highest level of agility ${q://QID1/ChoiceTextEntryValue} has achieved since recovery?

Training only - my dog has not competed in agility trials

Starters/Novice/Beginner

Advanced/Open/Intermediate

Masters/Elite/Excellent

International

Other - please specify

What best describes your perception of ${q://QID1/ChoiceTextEntryValue}'s return to agility performance?

Full return to pre-injury performance (no changes noticeable)

Satisfactory return to performance (few negative changes noticeable)

Moderate return to performance (many negative changes noticeable)

Unsatisfactory/extremely limited return to performance (drastic negative changes noticeable)

If you would like to provide additional details about your perception of performance changes, please do so here:

Dog demographics

You are almost finished! On the final pages of this survey we would like to collect some basic information about you and your dog. We'll start with ${q://QID1/ChoiceTextEntryValue}.

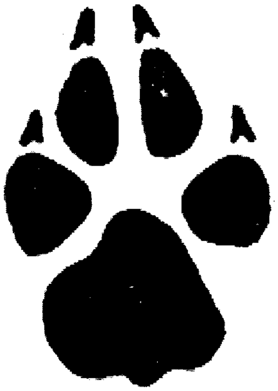

What is ${q://QID1/ChoiceTextEntryValue}'s sex?

Male

Female

What is ${q://QID1/ChoiceTextEntryValue}'s date of birth (approximate if unknown)? Enter date in the format mm/dd/yyyy

	Month	Day	Year
Date:			

What breed is ${q://QID1/ChoiceTextEntryValue}?

- Breed not listed
- Unknown/mixed
- Affenpinscher
- Afghan Hound
- Airedale Terrier
- Akita
- Alaskan Malamute
- American English Coonhound
- American Eskimo Dog
- American Foxhound

Is ${q://QID1/ChoiceTextEntryValue} intact or altered?

Yes, ${q://QID1/ChoiceTextEntryValue} was spayed or neutered

Yes, ${q://QID1/ChoiceTextEntryValue} was sterilized but with a hormone-sparing procedure (e.g., vasectomy, hysterectomy)

No, ${q://QID1/ChoiceTextEntryValue} is intact

What was the approximate date of the procedure? Enter date in the format mm/yyyy

	Month	Year
Date of procedure		

How old was ${q://QID1/ChoiceTextEntryValue} when spayed or neutered?

	Months	Years
${q://QID1/ChoiceTextEntryValue}'s age:		

What is ${q://QID1/ChoiceTextEntryValue}'s **height in inches** at the withers (shoulders) (approximate if unknown)?

What was ${q://QID1/ChoiceTextEntryValue}'s **weight in pounds** at the time of injury (approximate if unknown)?

How would you classify ${q://QID1/ChoiceTextEntryValue}'s weight?

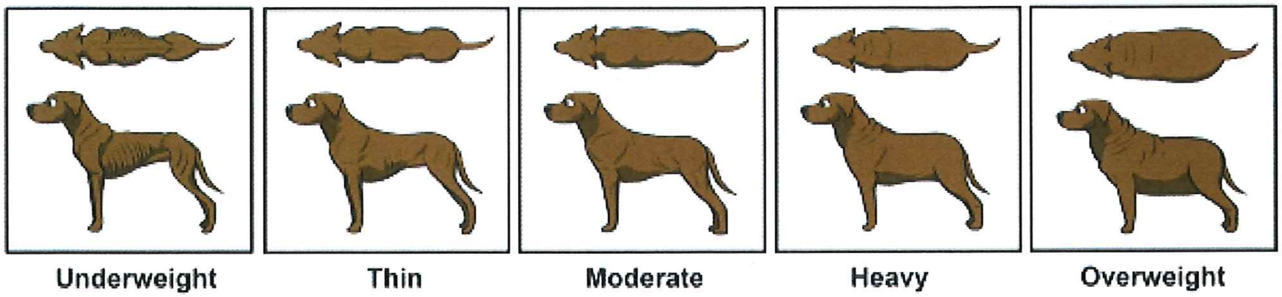

Reference the photo guide above in answering this question

Underweight *(spine, pelvis, and ribs prominent from a distance; lack of muscle mass; hollow rump; obvious waist; thin neck)*

Thin *(spine, pelvis, and ribs easily felt and visible on short-coated dogs; minimal body fat; obvious waist)*

Neither thin nor heavy *(spine, pelvis, and ribs easily felt; last few ribs may be visible, rounded rump; tucked-up waist)*

Heavy *(spine, pelvis, and ribs felt but never visible; flat abdomen; dog appears square from the side and above)*

Overweight *(spine, pelvis, and ribs difficult to feel; rolls of skin around neck and tail base; rounded abdomen; broad rump)*

Owner demographics

Finally, please tell us some basic information about yourself. You will then be asked for information consent, and this will be the last input page of the survey.

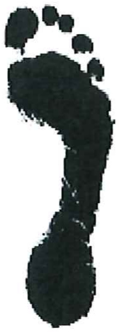

Approximately how many years have you been involved in agility?

With how many dogs have you participated in agility?

What is your gender?

Male

Female

Other

Prefer not to answer

Please select your age range from the options below:

Consent and identification

Your contact information (at minimum, please provide your name and a valid email address):

Name:

Email address:

Phone number:

City/Town:

State/Province:

Country:

May we contact you if we have additional questions about ${q://QID1/ChoiceTextEntryValue} related to this survey?

Yes

No

Do you agree to allow us access to your dog's agility performance records in conjunction with this study? (Note: this information is available from the sanctioning organizations and is required for full participation in the study)

Yes

No

Please provide ${q://QID1/ChoiceTextEntryValue}'s registration number for each agility venue in which he/she has competed:

AAC	
AKC	
ASCA	
CPE	
DOCNA	
NADAC	
UKC	
UKI	
USDAA	
Other	
Other	
Other	

Do you agree to allow us to contact your veterinarian for additional information related to the diagnosis and treatment of ${q://QID1/ChoiceTextEntryValue}'s injury that you described in this survey? (Note: this information is required for full participation in the study)

Yes

No

Please provide your veterinarian's contact information:

Veterinarian's Name:

Practice Name:

City/Town:

State/Province:

Country:

Email address:

Phone number:

Website:

Did an additional veterinarian participate in the diagnosis, treatment, or rehabilitation of ${q://QID1/ChoiceTextEntryValue}'s injury? If more than one additional veterinarian (two total) participated in treating ${q://QID1/ChoiceTextEntryValue}, you can enter their information in the open text box after this question.

Yes

No

Please provide the additional veterinarian's contact information:

Veterinarian's Name:

Practice Name:

City/Town:

State/Province:

Country:

Email address:

Phone number:

Website:

Is there any additional information you would like to provide

about ${q://QID1/ChoiceTextEntryValue}? If yes, please use this space:

Did you have any problems with the functionality of this survey?

No

Yes (please describe)

Powered by Qualtrics
