# Supplementary material for: Risk factors for cranial cruciate ligament rupture in dogs participating in canine agility
Source: BMC Vet Res. 2022 Jan 15;18:39. doi: 10.1186/s12917-022-03146-2 (PMC8760802; doi:10.1186/s12917-022-03146-2)
Supplement: Supplementary file 2 — Additional file 2. [file 12917_2022_3146_MOESM2_ESM.pdf]

Introduction

Purpose: Injuries to the cranial cruciate ligaments (CCL) in the knees of agility dogs are common. We recently completed a survey in which we collected information about more than 250 agility dogs with CCL injuries. Our goal is to identify risk factors for CCL injuries in agility dogs and to determine how likely injured dogs are to return to full athletic potential after various types of treatments. In order to accomplish these goals, we need data from healthy agility dogs that have never injured their cruciate ligatments to serve as a comparison (control) group.

This survey is intended to collect information related to dogs that are actively participating in agility competition and have never had a cruciate injury!

Eligibility: Any dog born between 1995-2014 that has competed in agility and *has never suffered from any knee injury or disease* is eligible to participate. All breeds/mixes, ages, and sexes of dogs are eligible. Owners completing this survey must be at least 18 years old.

Participation: Participation in this study is completely voluntary. No details about yourself or your dogs will be used beyond what you choose to provide or directly approve in your answers. You may quit the survey at any time and your answers to this survey will not be included in our research if you quit before completion.

Estimated Time: This survey takes approximately 10-15 minutes to complete. There will be detailed questions about your dog's agility training routine, as well as basic information about your dog's age, breed, sex, and size.

Who: The research team for this project includes Ms. Katherine Martucci (veterinary student at Washington State University), Dr. Debra Sellon (Professor of Equine Medicine at Washington State University), Dr. Denis Marcellin-Little (Professor of Small Animal Orthopedic Surgery at North Carolina State University), Dr. Michelle Powers (Small Animal Surgeon at Massachusetts Veterinary Referral Hospital), Dr. Kim Cullen (Research

Coordinator at Canines in Motion), and Sarah Fernandezlopez.

Ethics: This study was deemed exempt from review by the Institutional Review Board (IRB) at Washington State University.

If you have any questions, comments, or concerns, please contact Katherine Martucci at kmartucci@vetmed.wsu.edu.

What is the name of the dog for which you are submitting information?

If you currently have multiple dogs eligible for this study please submit **only one survey**.

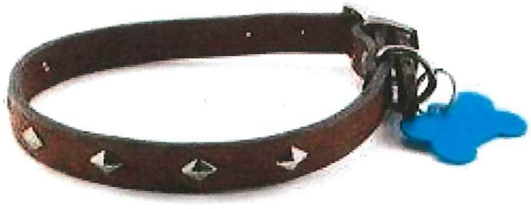

Has this dog ever been diagnosed with stifle (knee) or CCL (cranial cruciate ligament) disease?

*Note: we are only looking for participant dogs who **do not have** any history of stifle or CCL disease.*

No

Yes

Not sure

Agility Routine

We will start by asking some questions about ${q://QID1/ChoiceTextEntryValue}'s agility and activity routines. Please answer them to the best of your ability.

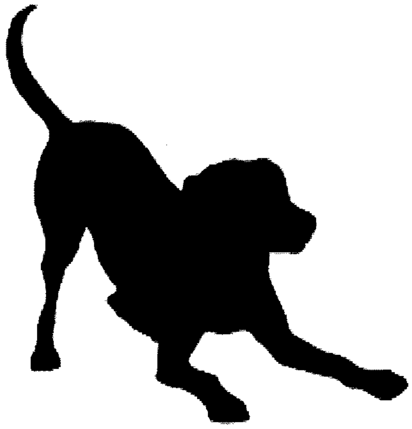

In which agility venues does ${q://QID1/ChoiceTextEntryValue} compete? Select all that apply.

AAC (Agility Association of Canada)

AKC (American Kennel Club)

ASCA (Australian Shepherd Club of America)

CPE (Canine Performance Events)

DOCNA (Dogs on Course in North America)

NADAC (North American Dog Agility Council)

UKC (United Kennel Club)

UKI (UK Agility International)

USDAA (United States Dog Agility Association)

International competition outside of North America

Other (please specify)

What is the highest level of agility that ${q://QID1/ChoiceTextEntryValue} has achieved in any venue?

Training only - my dog has not competed in agility trials

Starters/Novice/Beginner

Advanced/Open/Intermediate

Masters/Elite/Excellent

International

Other - please specify

On average, how many competitive agility events does ${q://QID1/ChoiceTextEntryValue} participate in annually?

1-5

6-10

11-15

16-20

More than 20

On average, how frequently does ${q://QID1/ChoiceTextEntryValue} train in agility?

Daily

3-6 times per week

1-2 times/week

Less than once per week

Has ${q://QID1/ChoiceTextEntryValue} participated (either training or competing) in any of the following sports besides agility? Please select all that apply.

Barn Hunt or Earth Dog

Conformation

Disc Dog

Dock Jumping

Flyball

Herding or Stock Dog

Hunting, Hunt Tests, or Field Trials

Lure Coursing or Racing

Mushing

Nosework

Obedience

Protection (e.g. Schutzhund, Ring Sport)

Rally

Weight Pull

Other, please specify

${q://QID1/ChoiceTextEntryValue} did not participate in any other sports

Do you do any weekly conditioning with ${q://QID1/ChoiceTextEntryValue} separate from sport training? Please indicate the frequency with which ${q://QID1/ChoiceTextEntryValue} participates in each of the following activities by grouping the activities into the categories below.

Click on a conditioning item in the left column and drag/drop it into the appropriate frequency box on the right.

Items

Core strength, balance, stretching, and body awareness exercises (e.g., wobble board, FitPaws, trick training)

10 / 10

Daily

3-4 times per week

1-2 times per week

Every other week

Monthly

Rarely

Never

If desired, use this space to further describe any regular conditioning activities you do with ${q://QID1/ChoiceTextEntryValue} separate from sport training. (optional)

Dog demographics

Great! Next we'd would like to collect some basic information about you and your dog. We'll start with ${q://QID1/ChoiceTextEntryValue}.

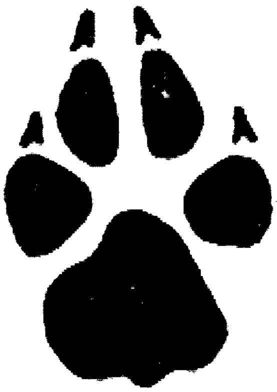

What is ${q://QID1/ChoiceTextEntryValue}'s sex?

Male

Female

What is ${q://QID1/ChoiceTextEntryValue}'s date of birth (approximate if unknown)? Enter date in the format mm/dd/yyyy

	Month	Day	Year
Date:			

What breed is ${q://QID1/ChoiceTextEntryValue}?

Breed not listed
Unknown/mixed
Affenpinscher
Afghan Hound
Airedale Terrier
Akita
Alaskan Malamute
American English Coonhound
American Eskimo Dog
American Foxhound

Is ${q://QID1/ChoiceTextEntryValue} intact or altered?

Yes, ${q://QID1/ChoiceTextEntryValue} was spayed or neutered

Yes, ${q://QID1/ChoiceTextEntryValue} was sterilized but with a hormone-sparing procedure (e.g., vasectomy, hysterectomy)

No, ${q://QID1/ChoiceTextEntryValue} is intact

What was the approximate date of the procedure? Enter date in the format mm/yyyy

	Month	Year
Date of procedure		

How old was ${q://QID1/ChoiceTextEntryValue} when spayed or neutered?

	Months	Years
${q://QID1/ChoiceTextEntryValue}'s age:		

What is ${q://QID1/ChoiceTextEntryValue}'s **height in inches** at the withers (shoulders) (approximate if unknown)?

What was ${q://QID1/ChoiceTextEntryValue}'s **weight in pounds** at the time of injury (approximate if unknown)?

How would you classify ${q://QID1/ChoiceTextEntryValue}'s weight?

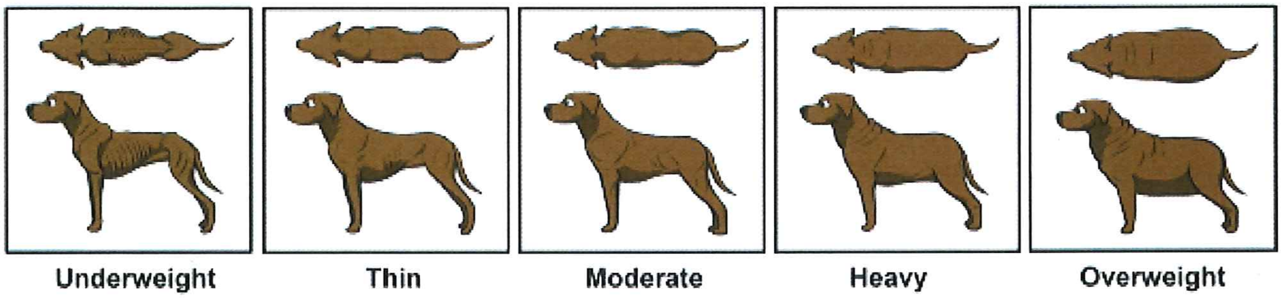

Reference the photo guide above in answering this question

Underweight *(spine, pelvis, and ribs prominent from a distance; lack of muscle mass; hollow rump; obvious waist; thin neck)*

Thin *(spine, pelvis, and ribs easily felt and visible on short-coated dogs; minimal body fat; obvious waist)*

Neither thin nor heavy *(spine, pelvis, and ribs easily felt; last few ribs may be visible, rounded rump; tucked-up waist)*

Heavy *(spine, pelvis, and ribs felt but never visible; flat abdomen; dog appears square from the side and above)*

Overweight *(spine, pelvis, and ribs difficult to feel; rolls of skin around neck and tail base; rounded abdomen; broad rump)*

Owner demographics

You are almost finished! Please tell us some basic information about yourself. The next page will be the final section of the survey.

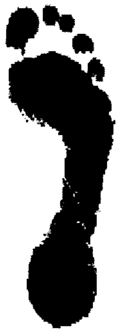

Approximately how many years have you been involved in agility?

With how many dogs have you participated in agility?

What is your gender?

Male

Female

Other

Prefer not to answer

Please select your age range from the options below:

Consent and identification

May we contact you if we have additional questions about ${q://QID1/ChoiceTextEntryValue} related to this survey?

Yes

No

Your contact information (at minimum, please provide your name and a valid email address):

Name:

Email address:

Phone number:

City/Town:

State/Province:

Country:

We would like to compare performance data for healthy dogs that have never had a cruciate ligament injury to the performance data for dogs that have had such injuries. With your permission this information will be requested from the sanctioning organizations and you will not be asked to provide it directly through this survey. Do you agree to allow us to access to your dog's agility performance records so that we can make this comparison?

Yes

No

Please provide ${q://QID1/ChoiceTextEntryValue}'s registration number for each agility venue in which he/she has competed:

AAC

AKC

ASCA

CPE

DOCNA

NADAC

UKC

UKI

USDAA

Other

Other

Other

OPTIONAL: Is there any additional information you would like to provide about ${q://QID1/ChoiceTextEntryValue}? If yes, please use this space:

OPTIONAL: Did you have any problems with the functionality of this survey?

No

Yes (please describe)

Thank you for participating in this research project!

Please hit the "next" button below to submit your response.

Powered by Qualtrics
